# The (in)visibility of deafness: Identity, stigma, quality of life and the potential role of totally implantable cochlear implants

**DOI:** 10.1111/hex.14060

**Published:** 2024-04-29

**Authors:** Chi Yhun Lo, Robyn Clay‐Williams, Beth Elks, Chris Warren, Frances Rapport

**Affiliations:** ^1^ Faculty of Medicine, Health and Human Sciences, Australian Institute of Health Innovation Macquarie University Sydney Australia; ^2^ Cochlear Limited Macquarie University Sydney Australia

**Keywords:** cochlear implants, disclosure, identity, quality of life, stigma, totally implantable cochlear implants

## Abstract

**Introduction:**

The disclosure of deafness is complex, given the historic and on‐going stigma associated with being deaf. The aim of this study was to explore how identity, stigma, and quality of life may be impacted when using cochlear implants (CIs) and totally implantable cochlear implants (TICIs). The physical difference between these two assistive listening devices is significant, given many CI users opt to hide their sound processor behind hair or headwear, in contrast to TICIs (an emerging technology) whereby all components are implanted internally and thus invisible.

**Methods:**

This qualitative study involved semistructured interviews and demographic questionnaires with 12 adult participants with more than 1 year of experience using their CI. Participants were recruited Australia‐wide through community organisations that support deaf and hard‐of‐hearing individuals. Interview transcripts were analysed thematically, with the themes generated through an inductive process, with consensus generated through group working with three members from the research team.

**Results:**

Four major themes were identified: (1) CI challenges; (2) The importance of social and support networks; (3) Identity and disclosure and (4) Concerns about TICIs. The underlying finding was centred around the construction of deaf identity. Participant attitudes were generally categorised as ‘Loud and proud’, with the recognition that displaying the CI was an extension of self, something to be proud of, and a means to normalise deafness; or ‘Out of sight and out of mind’, which sought to minimise the visibility of deafness. While both identities differed in how deafness is disclosed, they are fundamentally related to the same ideas of self‐agency and empowerment.

**Conclusion:**

TICIs present a novel opportunity—the ability for CI users to control the visibility of their deafness and thus control disclosure. This study explored the impact of stigma and categorised two core identities that CI users construct. Future directions include investigating *potential* CI candidates, to explore if TICIs may be a facilitator to CI uptake.

**Patient or Public Contribution:**

The semistructured interview guide was developed in consultation with adults with CIs. Feedback led to adjustments and improvement to the interview guide. In addition, F. R. has a lived experience with hearing loss, and C. Y. L. is an executive committee member for a nonprofit charity organisation that supports families that are D/deaf and hard‐of‐hearing.

## 1 INTRODUCTION

Deafness is an invisible disability—typically made visible by the external components of assistive devices such as cochlear implants (CIs) and hearing aids (HAs). Under normal circumstances, invisible or hidden disabilities are not apparent unless they are disclosed.^1^ However, the disclosure of deafness is complex. Hearing loss is fundamentally heterogeneous, with varied experiences and outcomes for individuals. Historically, cochlear implantation was recommended for adults with a severe to profound hearing loss, but contemporary recommendations indicate CIs are an effective treatment for adults with limited speech perception.^2^ Importantly, deaf and hard‐of‐hearing (DHH) identities are often positioned as being broadly bookended by the hearing and culturally deaf worlds—a cultural and sociolinguistic minority community typified by the use of sign language.^3^, ^4^ Taken together, DHH individuals contend with a delicate balancing act around the construction of their identity, place within the community, presentation of their identity, and the issue of disclosure.^5^

The visibility of deafness is highly stigmatised and associated with interrelated concepts, such as self‐perception (being perceived or labelled as disabled, impaired, incomplete and diminished), ageism and vanity,^6^ or the potential consequences of being discriminated against, which reinforce stigma (for a recent overview on stigma, see Ekberg and Hickson^7^). These feeling are expressed across all ages but with different contextual pressures. Younger adults generally contend with developing identities, the formation of social groups, wanting to fit into the hearing world and the development of social capital—broadly defined as connectedness and participation within social groups.^8^, ^9^, ^10^

However, late‐deafened adults are typically concerned with the maintenance of their ‘normal’ identity before hearing loss^11^ or reconciling multiple and potentially contradictory identities such as being hearing or being deaf.^12^, ^13^ Consequently, the stigma of deafness often leads to the denial and concealment of hearing loss; inaction and postponement to seek assistance; and social avoidance and isolation.^6^, ^14^ A recent study by Chang and Tucker^15^ investigated the perceptions of self‐stigma and socioemotional responses from adults who had acquired single‐sided deafness later in life. Their findings from 332 adults with unilateral CIs indicated that the more visible their CI was perceived to be, the more self‐stigma and social anxiety was experienced.

Concealment has always been a design consideration, whether it be for vacuum‐tube HAs worn on the body and torso in the 1930s; to the present, where invisible‐in‐canal HAs can be completely concealed.^16^ However, well before the electroacoustic age of HAs, attempts were made to hide bulky, mechanical devices such as ear‐trumpets. Camouflage techniques such as discreet placement within a flower bouquet or a decorative headband underscore the long history of stigma and attempts to conceal the visibility of deafness.^17^

CIs have also undergone their own pathway to miniaturisation and development. A typical contemporary CI consists of external and internal components. Visibly, the microphone, processor and battery are housed together and sit behind the ear (collectively referred to as the sound processor), cabled to a headpiece attached via magnet above and behind the skull. More recently, all‐in‐one sound processors are worn entirely off the ear (e.g., Cochlear Kanso™ and MED‐EL Rondo™). In these systems, all the components are integrated into a single unit with no external cabling. While all‐in‐one systems have received positive subjective appraisals for comfort and cosmetics from user surveys,^18^ attitudes on stigma from users have yet to be appraised.

Alternatively, instead of utilising strategies of concealment, some users modify and customise their HAs and CIs with stickers and jewellery to draw attention to them. Aesthetic customisation may change attitudes and counteract stigma by being visually appealing and fashionable, and this act of self‐expression may promote feelings of agency, empowerment, confidence and pride.^19^, ^20^ One noted benefit is through communication signalling, in which bystanders may more easily identify the user's status as DHH, potentially improving understanding of communication needs or responses to communication needs from others.^20^

Totally implantable cochlear implants (TICIs) are a next‐generation CI technology that are not currently commercially available but have been trialled for viability.^21^, ^22^ TICIs represent a significant paradigm shift, as all elements such as microphones and batteries are positioned internally, under the skin and thus completely hidden. TICIs are anticipated to provide cosmetic benefits, reduce stigma, improve convenience, and accommodate headwear use.^23^, ^24^

However, research into the aesthetic and cosmetic concerns around CIs and its association with identity, stigma, and quality of life is extremely limited. An additional consideration is the widespread underutilisation of CIs^25^, ^26^ and the extent that physical and cosmetic characteristics are a barrier to improving hearing health.^9^, ^14^, ^27^, ^28^ Taken together, an exploration of the relative importance of cosmetic concerns and underlying advantages of CIs (including TICIs) is warranted and forms the basis of the present study.

The conceptualisation of TICIs provides the opportunity to reimagine the CI as invisible. Thus, the aim of this study was to understand the potential impact of TICIs on the broad issues of identity and disclosure, its role in stigma and quality of life. The primary objective was to explore how these would differ between CIs and TICIs, to better understand the relative advantages and disadvantages of each system. The secondary objective was to assess the use of TICIs for daily activity, such as work and recreation, and general usability.

## 2 MATERIALS AND METHODS

### 2.1 Design

This was a qualitative methods study set in Sydney, New South Wales, Australia. Data was collected in 2021–2022. Participants completed (1) a demographic questionnaire and (2) individual semistructured interviews. See the published open‐access protocol article for additional detail.^29^

### 2.2 Participants

Participants were recruited Australia‐wide through online flyers distributed to community organisations such as Hear For You (a charity organisation that supports and mentors young DHH adults), Hearing Matters Australia (an advocacy organisation dedicated to helping Australians with hearing loss) and CICADA Australia (a volunteer support group for CI users and potential CI candidates).

All participants were (1) adults aged 18 years and older, (2) a CI user with more than 1 year of experience using their device, (3) proficient in English and (4) in possession of sufficient cognitive capacity to complete the demographic questionnaire and (5) able to engage effectively in the semistructured interview as determined by the interviewer.

The participant recruitment strategy resulted in participants with a broad range of characteristics across age, gender, bilingual Auslan use, employment status, and pre‐/postlingual implantation. The protocol article for this study estimated between 10 and 15 participants would be required to reach data saturation.^29^ A total of 12 participants (7 women, 5 men) were recruited and all of them had completed tertiary‐level education (certificate/diploma or higher), with the majority being employed, and living in a major Australian city. Eight participants were postlingually deafened, and four participants were bilingual English/Auslan (Australian Sign Language) users. See Table 1 for a demographic summary, Table 2 for an audiological summary and Table 3 for individual participant characteristics.

**Table 1:** Demographic summary.

	Count
Age	
18–34	3
35–49	3
50–64	4
65–74	2
Gender	
Woman	7
Man	5
Relationship status	
In a married/de facto relationship	6
Not in a married/de facto relationship	6
Children and dependents	
Yes	3
No	9
Household income	
$0–$39,000	1
$39,001–$98,000	6
$98,001 or more	4
Prefer not to say	1
Education	
Certificate, diploma, advanced diploma, or associate degree	5
Bachelor's (honours) degree, graduate certificate/diploma	4
Master's degree	2
Doctoral degree	1
Employment	
Full‐time employment	5
Part‐time employment	2
Casual employment	3
Retired	2
Private health insurance	
Yes	11
No	1
Remoteness structure	
Major city	10
Inner regional	1
Outer regional	1
Language use	
English	7
English and Auslan	4
English and Indonesian	1
Comorbidities	
None	5
One	4
Two	3

**Table 2:** Audiological data.

	Count
Age of hearing loss diagnosis	7
0–5	
6–12	2
13–17	1
18–34	0
35–49	1
50–64	1
Pre‐ or postlingual aiding/implantation	
Prelingual (≤3 years old)	4
Postlingual (>3 years old)	8
Aetiology of hearing loss	
Genetic	4
Disease (e.g., rubella, meningitis)	2
Ototoxic drugs	1
Otosclerosis	1
Ménière's Disease	1
Unknown	3
Level of hearing loss (left ear)	
Mild	0
Moderately severe	2
Severe	1
Profound	4
Complete	5
Level of hearing loss (right ear)	
Mild	1
Moderately severe	0
Severe	3
Profound	4
Complete	4
Experience using a device (left ear), years	
0–5	5
6–10	3
11–15	2
16–20	0
More than 20	2
Experience using a device (right ear), years	
0–5	3
6–10	2
11–15	2
16–20	0
More than 20	5

**Table 3:** Individual participant characteristics.

ID	Age bracket (years)	Gender	Relationship status	Children and dependents	Household income	Education	Employment	Private health insurance	State and remoteness	Language use	Comorbidities	Pre‐ or postlingual aiding/implantation	Aetiology	Degree of hearing loss	Device experience (years)
1	35–49	Man	Married/de facto relationship	Yes	$39,001–$98,000	Bachelor's (honours) degree, graduate certificate/diploma	Full‐time	No	VIC (major city)	English	None	Post	Otosclerosis	L: Complete R: Profound	L: 4 R: 5
2	50–64	Woman	Married/de facto relationship	No	$98,001 or more	Master's degree	Part‐time	Yes	VIC (major city)	English	None	Post	Genetic	L: Moderately‐severe R: Complete	L: 9 R: 10
3	65–74	Woman	Married/de facto relationship	No	$39,001–$98,000	Doctoral degree	Retired	Yes	NSW (inner regional)	English	None	Post	Unknown	L: Complete R: Complete	L: 6 R: 4
4	65–74	Man	Married/de facto relationship	No	$39,001–$98,000	Certificate, diploma, advanced diploma, or associate degree	Retired	Yes	NSW (major city)	English	2	Post	Ménière's disease	L: Complete R: Mild	L: 12 R: 0
5	50–64	Woman	Not married/de facto relationship	No	$98,001 or more	Master's degree	Full‐time	Yes	VIC (major city)	English and Auslan	1	Pre	Disease (e.g., rubella, meningitis)	L: Profound R: Severe	L: 0 R: 14
6	18–34	Woman	Not married/de facto relationship	No	$0–$39,000	Bachelor's (honours) degree, graduate certificate/diploma	Casual	Yes	NSW (major city)	English and Auslan	1	Pre	Genetic	L: Profound R: Profound	L: 15 R: 23
7	35–49	Woman	Not married/de facto relationship	No	Prefer not to say	Bachelor's (honours) degree, graduate certificate/diploma	Casual	Yes	NSW (major city)	English and Indonesian	None	Post	Ototoxic drugs	L: Severe R: Severe	L: 26 R: 27
8	35–49	Woman	Married/de facto relationship	Yes	$98,001 or more	Certificate, diploma, advanced diploma, or associate degree	Part‐time	Yes	SA (major city)	English	1	Pre	Genetic	L: Moderately‐severe R: Profound	L: 2 L: 35
9	18–34	Woman	Not married/de facto relationship	No	$39,001–$98,000	Certificate, diploma, advanced diploma, or associate degree	Casual	Yes	NSW (major city)	English and Auslan	1	Pre	Genetic	L: Complete R: Severe	L: 24 R: 25
10	50–64	Man	Not married/de facto relationship	No	$39,001–$98,000	Certificate, diploma, advanced diploma, or associate degree	Full‐time	Yes	NSW (major city)	English	None	Pre	Unknown	L: Profound R: Complete	L: 4 R: 11
11	50–64	Man	Not married/de facto relationship	No	$39,001– $98,000	Bachelor's (honours) degree, graduate certificate/diploma	Full‐time	Yes	NT (outer regional)	English	2	Pre	Disease (e.g., rubella, meningitis)	L: Complete R: Complete	L: 4 R: 49
12	18–34	Man	Married/de facto relationship	Yes	$98,001 or more	Certificate, diploma, advanced diploma, or associate degree	Full‐time	Yes	QLD (major city)	English and Auslan	2	Post	Unknown	L: Profound R: Profound	L: 5 R: 6

Abbreviations: L, left; R, right.

### 2.3 Semistructured interview

Before the interview, participants received a preinterview information sheet. This one‐page document summarised and clarified key terms such as ‘discretion’ (defined in this study as how unobtrusive or subtle a CI appears), provided close‐up photographic examples of an all‐in‐one system being used by a man and a woman, and a conceptual schematic of a TICI. The purpose of this preinterview information sheet was to provide participants with a frame of reference before the interview.

The interviewer (redacted for peer review) had considerable experience on the topic of deafness and CIs through research expertise; education and training such as Auslan certification and Deaf Awareness Training; and through community‐focused work, serving as an executive committee member for a nonprofit charity organisation that supports families that are D/deaf and hard‐of‐hearing.

The semistructured interview schedule was developed by a team of researchers and an adult with a CI, in line with the current literature on DHH identity, need, hearing healthcare service experience and stigma. The following topics were broadly examined: (1) benefits and challenges associated with participants’ current CI use; (2) the impact of discreet CIs such as TICIs on communication, motivation, social interactions, and quality of life; (3) hearing healthcare pathways; (4) trust, influence, and relationship with healthcare providers and stakeholders and (5) learning about CIs and information access. See Supporting Information S1: Material 1 for the semistructured interview schedule.

### 2.4 Procedures

All participants provided written informed consent before data collection. Participants were advised that all quotes would be deidentified and names replaced with pseudonyms. Participant comfort and wellbeing was prioritised and reinforced. As such, participants were reminded that their participation was completely voluntary and that they had the right to withdraw from the study at any time, without giving a reason for doing so and with no effect on their on‐going healthcare provision.

All data collection materials (demographic questionnaire and interview) were disseminated online for ease of participant access and designed to be completed in approximately 1.5 h in total (30 min for the demographic questionnaire and an hour allocated for the interview). The semistructured interviews were conducted via the videoconference application Zoom with on‐screen captioning enabled. Interviews were audio recorded, deidentified, and transcribed verbatim by an external transcription service. Participants were provided with an AUD50 e‐gift card as a token of appreciation for their time and effort.

### 2.5 Data analysis

Demographic characteristics were analysed using descriptive statistics in IBM SPSS Statistics for Windows, Version 27.0.^30^ Transcripts and researcher fieldnotes from the semistructured interviews were analysed using a six‐phase approach to thematic analysis which required: (1) familiarisation with the data, (2) generation of initial codes, (3) searching for themes, (4) reviewing themes, (5) defining and naming themes and (6) producing a data findings report.^31^

Thematic analysis was conducted with the support of a group‐working technique in which ideas were shared inductively, as analysis progressed and discussed through fortnightly group working sessions, to ensure consensus opinion on key issues arising, thematic patterns, and incongruities.^27^, ^29^, ^32^ Three qualitative expert analysts (C. Y. L., R. C.‐W., F. R.) contributed, and their prior experience ensured this collaborative approach and process was robust and rigorous. Multiple discussions worked towards a final thematic framework. Coding and analysis of the demographic data, fieldnotes and transcripts were completed using NVivo (released in March 2020).^33^

## 3 RESULTS

### 3.1 Thematic analysis of semistructured interviews

Four key themes and their concomitant categories were generated that relate to the broad issues of identity and disclosure, stigma, and quality of life: (1) CI challenges; (2) The importance of social and support networks; (3) Identity and disclosure and (4) Concerns about TICIs. Most of the data was identified within these themes, indicating data saturation had been achieved. The first three themes are primarily in respect to existing CI technology, but any relevance to TICIs is clearly labelled. Quotations from the transcribed interviews were deidentified and labelled in this article by participants (P1–P12).

### 3.2 Theme 1: CI challenges

Overall, the CI was seen to be an overwhelmingly positive, life‐changing experience for all participants with significant benefit for speech perception, psychosocial wellbeing, and overall quality of life. However, many CI challenges remain, such as stigma and discrimination, dislodging or losing a CI sound processor and usability.

#### 3.2.1 Stigma and discrimination

Nearly all participants noted they had experienced some form of stigma and discrimination that was associated with both deafness and the visibility of deafness (noting many participants were HA users before becoming CI users). This took many forms and varied across a wide spectrum of severity and contexts. It included microaggressions, being devalued and dehumanised, and facing resistance to social inclusivity.

You don't hear somebody, and they look at you, see the CIs and speak to you as if you're retarded… Or people choose to speak to your partner instead. Some people are just uneasy or unsure how to approach you. (P12)

Pity was commonly received from strangers, contributing to the stigmatising idea of being a burden, and further reiterating the general public's misunderstanding of the experience of deafness.

I get a sense of pity… Don't give me that, because you weren't there when it happened, you didn't do anything. Having a CI doesn't mean I've got it bad. (P6)

One participant noted that the visibility of hearing devices and associated stigma was so profound that they considered it was a barrier to device uptake.

I have many friends who have hearing problems and they won't even get a basic hearing aid, because of the ugliness, the perception that you have a hearing disability, and that you're old and senile and everything else. So, they're putting it off… (P3)

Participants recognised that stigma and discrimination were particularly prevalent in the workplace, with employers, supervisors and colleagues often not understanding or addressing their specific needs, or at worst, simply ignoring them.

One of my biggest challenges… I have a CI, and this is how I would like you to communicate to me—they wouldn't acknowledge that… I'm able to do the work but if I need to communicate to somebody… (P6)

While many participants were happy in their present occupation (noting that many were working in deaf/hearing organisations), many had experiences of not being adequately supported, with work interactions often impactful and harmful.

I think it affected me more in a work setting because I was surrounded by a few people that didn't approach it in the right way. It impacted me more than I ever told anyone. (P8)

The unique context of attending workplace interviews also brought about concerns of how pre‐existing notions of deafness may negatively affect job prospects.

The stigma could be felt when I went for some recent interviews… The CIs enabled me to communicate on phone interviews and I was able to get face‐to‐face interviews. However, I could see hesitation on their face when they knew about my hearing loss at the end of the interview. (P7)

The perception that the CI itself was a requirement to prove and authenticate deafness to the general public was not an uncommon sentiment. This was closely aligned to themes around identity and communication signalling (discussed at greater length in Theme 3: Identity and disclosure).

Growing up, I was a bit insecure of having my CI because it showed that I'm deaf and I felt people didn't want to talk to me because they thought I'd be difficult to interact with. If it's hidden, maybe there will be a different sense of confidence. (P6)

#### 3.2.2 Dislodging or losing a CI sound processor

Dislodging or losing a CI sound processor was a common occurrence, which led to an increased level of awareness, care and anxiety.

I'm just a little bit careful. It becomes second nature. Getting onto a train, before I step from the platform, I put my hand up here [raised hand below ear] just in case somebody bumps me, and it falls off. (P1)

#### 3.2.3 Usability

Participants were often dissatisfied with the usability of the CI, particularly the spontaneity that comes with social activity. These seemingly innocuous ‘little things’ were often perceived as compromises that impacted day‐to‐day activities and quality of life.

They're very important things that niggle on you, like a tiny toothache or something that can take over your life. I'm very grateful for what I have, but it's not without its stresses… I said to my husband, ‘This is just too hard. I just want to be able to go and have a swim, and I've got to do all of this preparation.’ So, in the end I've given up. (P3)

Participants were optimistic that usability was one area that TICIs would appreciably improve.

I think it would make a world of difference. Hear without the hassle of those annoying things. Sometimes those annoying things you might think are small, but when there are lots of little annoying things… (P8)

### 3.3 Theme 2: The importance of social and support networks

Being diagnosed with a deafness, parsing information and going through the decision‐making process was generally overwhelming. For most participants, access to a robust support network was critical for achieving successful outcomes with CIs. The gateway to this support network was typically through their audiologist and otolaryngologist, and in particular, finding the right team or organisation that understood their specific needs.

The audiologist knew I was desperate because I'm so involved in music. I thought if I lose my music capacity, then I just wouldn't know who I was. And they were beautiful to me. They put me in touch with musicians who had CIs, so that I could just contact them and share my woes, my scares, the things I was frightened about, and learn from their experience. (P3)

Many participants were influenced to undergo CI surgery after having discussions with a CI user, emphasising the importance of sharing concerns with someone with a lived experience.

I wouldn't say it was an audiologist or anyone specific, apart from maybe this person who had gone through it themselves that made me go yeah—I'm going to do it. (P8)

Friends and family were invaluable, and often relied upon for advice and support. Of particular note was the importance of being able to connect with others who shared the experience of deafness, and long‐term friendships were in part defined by this ability to understand and empathise with the situation and circumstances.

My deaf friends really became invaluable during that season [of depression] as people I could talk to, who wouldn't try to give silly solutions or tell me that it wasn't that bad… (P9)

The following example highlights the importance of friends and family as intermediaries in a hearing world and the consequent anxiety and stress with managing an appointment alone. The extended period of having to maintain vigilance and attention is likely a contributor to fatigue. Typically, this participant would attend medical appointments with her mother as social support.

This time I didn't have somebody else to be my ears. Going to appointments alone I feel anxious because I don't know if I would be able to hear, or feel stupid, or look stupid in front of them… Or when do I bring up, ‘Hi, sorry, I'm deaf…’? I would have anxiety waiting for my name to be heard. Every time a person comes out, I have my hearing to full attention to see what they say but they were wearing masks. (P6)

### 3.4 Theme 3: Identity and disclosure

Identity and the disclosure of identity was the present study's central theme. Participants' deaf identity was highly individual, but two common and divergent groupings were labelled: ‘Loud and proud’ or ‘Out of sight and out of mind’.

#### 3.4.1 Loud and proud

For participants aligned with the ‘Loud and Proud’ identity, the CI was often perceived as an extension of themselves, and thus something to celebrate and be proud of.

I don't even know it's there, it's just part of me. To be honest, I wear it loud and proud. I'm quite happy to wear it. (P10)

That visibility—that shows me what I am… I just can't imagine life not being a deaf person. I'm proud I was born deaf. (P11)

#### 3.4.2 Out of sight and out of mind

The visibility of the CI often created feelings of shame, embarrassment, and disempowerment for the participants aligned with the ‘Out of sight and out of mind’ identity. The most common strategy was to hide the CI underneath hair. In particular, the visibility of the CI triggered a powerful response from one participant who had held strong, negative views towards the aesthetics of HAs and CIs.

It's awful going around with this great big advertisement on your head. There is nothing pretty about any hearing aid or any cochlear processor that I have ever seen in my life… It's an advertisement to the world that you have a hearing disability, and that you're old and senile, and everything else… Some people wear them proudly and they put jewellery all over them and do all sorts of pretty things, but for me, it was just shame and embarrassment… Out of sight is out of mind. So, if people can't see it, then I feel quite confident… I just want to be like a normal person, like everybody else. (P3)

However, for others, the purpose of hiding a CI was far more nuanced than simply avoiding stigma and negative perceptions. In these cases, the act of hiding a CI provided participants the ability to choose when to disclose their hearing status without prejudgement.

It's just always been subconscious, because even when I do have my hairdo up, I still put it down because I just don't want it to be seen… But am I really that ashamed of it or is it a part of me that I don't want to invite people to know?… I don't like having the CI showing because it notifies others that I'm a deaf person and I'm giving consent for them to make an assumption. People would be like, oh, the CI, let's talk about it. No—I get to start first. (P6)

#### 3.4.3 Flexible, contextual and developing identities

Identity was not necessarily a fixed construct. Many of the participants expressed how their conception of topics such as deafness, disability and identity had developed through experience and time. Much of this was tied to how participants' reconciled attitudes around acceptance or denial of their deafness.

I spent a very long period of my life wanting to hide the fact that I had hearing loss. I went through the majority of my working life really struggling in terms of my emotions and feeling of self‐worth. And I think a lot of it really came down to this whole denial of hearing loss and wanting to hide and not wanting to let people know. I've actually done an about‐face. I really couldn't care less whether people see my devices or not… (P1)

#### 3.4.4 Disclosure

How participants displayed their identity and disclosed their deafness was also context dependent. Participants often expressed hesitation disclosing their deafness amongst strangers and in the workplace, particularly during the interview and hiring process.

I think when it comes to learning—I'm a deaf person. I can't hear things, I will miss out on things, I need all of the help that I can get, note taker, whatever. But when it comes to social life… I want to do this myself; I want to be selective of what I say about being the deaf person. I don't want to go out to everyone that I meet and be like, ‘Hi, I'm deaf’. I think it's because I want to have an identity that doesn't say, oh, she's that deaf girl. (P6)

I don't have any issues with it. In saying that if I were to go for a job interview tomorrow, to be honest, I probably would cover it. Discrimination unfortunately… It is still out there… (P8)

One participant had developed sophisticated and nuanced strategies to organically disclose their deafness and accommodations.

In every situation, what's the right timing to get the best result? Because I want it to be after I've gotten enough chance to show that I can communicate, because if you say it too early, people kind of freak out and don't know how to talk to me anymore… But I don't want to leave it too late and then be struggling to communicate. Often there will come a natural point where I'll need to ask for someone to repeat something. And then I can insert it then, and it feels quite natural… Or if I'm sitting down for an interview, and they leave the door open and there's ambient noise coming in. I might say, ‘Sorry, do you mind if we close the door? I'm deaf’. (P9)

#### 3.4.5 Communication signalling and the burden of proof

For some participants, the visible display of the CI was used as an important and effective communication signal, to prove or ‘authenticate’ deafness and as a useful social marker.

I shouldn't have to prove I'm deaf, but it does help people understand that I really am deaf when I can point to something and say, look, I'm deaf, I have a cochlear. (P9)

They'll have a CI, and you look at each other and you kind of give each other that kind of nod as to say, hey, we're part of a secret society. (P1)

#### 3.4.6 Advocacy

A common enthusiastic sentiment was to give back to the community through advocacy. Interestingly, while the majority of participants had received excellent support during their process of learning about CIs, undergoing surgery, and subsequent rehabilitation; even participants who did not receive adequate support were motivated to become advocates, to ensure future CI candidates had a better experience than their own.

It's very easy for people that have hearing loss to get overwhelmed by it. It can be all‐consuming sometimes. You feel like you're alone in this world and nobody understands what it's like and I remember I went through many, many years like that. I think the biggest lesson I learned is that I can't expect people to understand what it's like unless they understand. And I'm in this unique position to be able to give that understanding. (P1)

### 3.5 Theme 4: Concerns about TICIs

Given that TICIs are not a commercially available device, participants were presented with some basic information and context about TICIs via the preinterview information sheet. If queried, the interviewer (C. Y. L.) consistently reiterated they could not provide specific details about TICIs beyond the preinterview information sheet, but participants' concerns were nonetheless acknowledged. Within this framework, participants had specific concerns around the potential use of TICIs including safety, such as the likelihood of faults and how upgrading would work.

It's the safety issues that does concern me a little bit—batteries in the skull. If it was in any other parts of the body, they're more accessible. (P4)

Would I be able to upgrade as technology upgrades? I like the fluidity of being able to get new processors when new stuff comes out. (P9)

Positively, participants anticipated TICIs would provide numerous benefits such as ‘always‐on listening’ that could improve safety, better access to activities (particularly swimming and activities that required helmet use), and not losing or dislodging the CI.

If I take my devices out, I don't hear anything. When I'm going to bed at night or in the shower, all those things, and somebody's calling me, I don't hear. So that would be one benefit I suppose, for that hidden one. (P1)

I want to get it because I'm a swimmer. It's probably less to do with the discreetness about it… But to be able to go in the water and hear, because I get quite a bit unnerved… Sometimes if I go late, I get a tap on the shoulder, because I haven't heard the announcement to get out of the pool … it would be quite an amazing benefit. (P10)

## 4 DISCUSSION

This qualitative study provides empirical data from adult CI users in relation to the impact and role of CIs and the hypothetical role of TICIs in their lives. The thematic analysis of the semistructured interviews revealed four major themes: (1) Cochlear implant challenges; (2) The importance of social and support networks; (3) Identity and disclosure and (4) Concerns about TICI. To the best of our knowledge, this is the first qualitative examination of aesthetic and cosmetic considerations of CIs with respect to issues of identity, stigma, and quality of life.

The underlying theme of the present study was centred around the construction of deaf identity. The two prevalent identities were either ‘Loud and proud’ or ‘Out of sight and out of mind’. These labels are closely aligned to ‘Asserting oneself’ and ‘Avoiding/ignoring’, themes that were identified in a qualitative study that examined how DHH adults responded to perceived stigma in the United States.^11^

At face value, the ‘Loud and proud’ and ‘Out of sight and out of mind’ identities appear to be oppositional. However, we propose that these are both fundamentally based around strategies that provide agency and empowerment but differ by the process in which disclosure occurs. First, our data supports the idea that participants were autonomous, self‐aware of their capacities and would often contextually limit their interactions. For example, avoiding large group events, being aware of the optimal seating placement in a restaurant, and preferring smaller gatherings; avoiding unfamiliar music, but being receptive to familiar music, or supplementing it with a musical score.

Second, all participants had experienced some level of stigma and discrimination because of their deafness. The words and behaviours directed towards participants could be particularly vitriolic (e.g., being described as intellectually disabled, being pitied, being ignored). Our overall findings are in accordance with the themes identified by Lash and Helme^11^: feelings of pity, not being worth others' time, being labelled as ‘not normal’, hearing loss limiting capabilities and intelligence and deafness being different than other disabilities. This last theme touched upon the idea that the invisibility (or the ability to hide) deafness made it particularly susceptible to being ignored and misunderstood.^11^

Third, being ‘Out of sight and out of mind’ may be an effective strategy to manage stigma, by hiding deafness. Unfortunately, this may have additional consequences that generate the conditions for loneliness and isolation.^11^, ^34^ Many participants were happy to disclose their deafness and communication needs, but rarely in the first instance, as they did not want to be pigeonholed as being ‘the deaf person’. Importantly, the present study identified that the CI users within this identity grouping would greatly appreciate the empowerment provided by TICIs, as the invisibility of the device would provide them the opportunity to disclose their deafness on their own terms.

This contrasts to the ‘Loud and proud’ identity, whereby disclosure of deafness *is* the apparent and intentional visibility of the CI. There are inherent advantages to this approach such as communication signalling, as ‘proof’ of deafness (typically in the context of conveying hearing status to strangers), as a group marker, to be assertive, and to normalise the existence of deafness.^5^, ^11^, ^20^ Taken together, we contend the two identities are fundamentally derived from strategies related to self‐agency and empowerment.

Importantly, while this study identified two broad identities that relate to the visibility of the CI (‘Loud and proud’ and ‘Out of sight and out of mind’), this categorisation is not intended to be definitive nor strictly binary. There was also no evidence that either prelingual or postlingual deafness was associated with either identity. It is important to acknowledge the existence of myriad identities on a spectrum that reflect the complexity of deafness, the contextual nature of identity, and the fluidity of identity.^35^, ^36^ Importantly, this ties into the ‘possibility model’—understanding CI experiences through the lens of possibility, rather than that of a traditional (and potentially limiting) role model.^37^ Our data supports the theoretical and empirical perspectives that the construction of identity is an on‐going negotiation that occurs across all layers of identity and is ultimately bound by wanting to belong.^36^, ^38^

Generally, participants did not want their first impression to be about their deafness in place of their personhood, particularly in the context of a work interview. Hence, participants typically preferred to disclose their deafness and discuss their communication needs after securing employment. As others have noted, this study also found participants would hide their CI behind their hair for interviews, informed by their experience with stigma, discrimination and the perception that without so doing, they would be seen to be less competent or unable to fulfil the requirements of the job.^5^, ^39^ Data from the present study found some participants had sophisticated techniques to organically integrate communication needs into the conversation, such as pointing out noise (e.g., ‘Could you close that door, I'm deaf’), or reaching a point in conversation whereby asking someone to repeat a question could be used as the starting point to introduce their deafness (e.g., ‘Could you repeat that? I'm hard‐of‐hearing’).

Prior research has indicated that employers can be reluctant to make accommodations for DHH employees.^39^ The present study data echoes this, indicating that even when workplaces are made aware of people's communication needs, they can simply be ignored. This study highlights how workplace discrimination can become entrenched discriminatory practices which may contribute to the reluctance DHH workers have to request accommodations. This remains an on‐going barrier that contributes to the underemployment of DHH adults.^40^, ^41^ Overall, while many participants noted that TICIs would be beneficial in situations such as interviews, its effect on the negotiation of accommodation and impact on broad workplace outcomes is an open question.

CI users recognised their unique position as advocates to authentically share their experience to improve outcomes for others. A significant turning point to undergo implantation was often through discussions with experienced CI users. Hence, participants were motivated to continue this cycle of support and encourage the adoption of CI devices. Similar to findings by Profita et al.,^20^ interactions with children were described by participants aligned with ‘Loud and Proud’ as opportunities to have fun with their CI by placing bright stickers on their device. However, identities did not necessarily limit advocacy, they simply affected how the CI was displayed and disclosed. For example, P6 was aligned with ‘Out of sight and out of mind’ and was also engaged in a mentoring programme that supported DHH youth.

This study has several limitations. The cohort was drawn from a Western, Educated, Industrialized, Rich, and Democratic society and subject to all the criticisms that stem from this specific background.^42^ In particular, attitudes around disability and stigma differ significantly across culture and are culturally specific.^43^ While the sample size of 12 participants was sufficient to generate data saturation of key themes, a larger and more culturally and linguistically diverse cohort may better account for the heterogeneous nature of deafness. Swimming activities were a common theme, but this may be culturally specific, given the relative importance of swimming in Australia. The ratio of participants that were White (11/12) and tertiary‐level educated (12/12) was also not reflective of the general Australian population. TICIs are also not a commercially available device, thus the ideas expressed by participants on TICIs were inherently conceptual. However, the provision of the preinterview sheet ensured there was some grounding and context provided.

This study examined perspectives from *experienced* CI users, but future studies should also examine *potential* CI candidates, to further understand the impact of CI aesthetics as a barrier for CI uptake. The systematic assessment of identity and stigma for CI users across a broad range of lifestyles and contexts (e.g., vocational, educational, recreational, etc.) is also recommended to best represent the dynamic nature of identity. In summary, a broader and more intersectional cohort of potential CI candidates and current CI users across different cultures should be examined in future studies.

## 5 CONCLUSION

This study utilised TICIs as a thought‐provoking device to examine the impact of the (in)visibility of deafness. The data supports CI users falling into two broad identities: ‘Loud and proud’ and ‘Out of sight and out of mind’. While both enact and disclose their identities in vastly different ways, fundamentally they address the same issue of stigma and are intended to empower the individual. The usability of TICIs was rated positively, with participants anticipating benefits for safety, wearing headgear, and not losing or dislodging a CI sound processor; although there were concerns around how faults would be rectified. Compared to a contemporary CI, a significant advantage of TICIs is the flexibility to present or display an identity, which may be useful as identities evolve, and also allow identities to be enacted differently across a range of contexts and situations. The specific importance of hiding deafness during work interviews was also noted and is highly relevant given the underemployment of DHH adults. Finally, the ability to control the visibility of deafness and thus control disclosure was seen as highly desirable and empowering and would likely reduce the burden of stigma for participants aligned with the ‘Out of sight and out of mind’ identity.

## AUTHOR CONTRIBUTIONS

**Chi Yhun Lo**: Writing—original draft; writing—review and editing; methodology; conceptualisation; investigation; data curation; formal analysis; validation. **Robyn Clay‐Williams**: Methodology; conceptualisation; writing—review and editing; formal analysis; supervision; validation. **Beth Elks**: Conceptualisation; writing—review and editing. **Chris Warren**: Writing—review and editing; conceptualisation. **Frances Rapport**: Funding acquisition; writing—review and editing; conceptualisation; methodology; project administration; formal analysis; supervision; validation.

## CONFLICT OF INTEREST STATEMENT

Chi Yhun Lo has provided consulting services for Cochlear Limited on unrelated projects in the past. Beth Elks is, and Chris Warren was, employed by Cochlear Limited. They were involved in the conceptualisation, and writing—review and editing. They were not involved in the investigation or methodology. The remaining authors declare no conflict of interest.

## ETHICS STATEMENT

This study was granted ethical approval from Macquarie University Human Research Ethics Committee (HREC: 520211056232432) and meets the requirements set out in the National Statement on Ethical Conduct in Human Research. All participants provided written informed consent before data collection.

## Supporting information

Supporting information.

## Data Availability

The data that support the findings of this study are available on request from the corresponding author. The data are not publicly available due to privacy or ethical restrictions.
