# Supplementary material for: The (in)visibility of deafness: Identity, stigma, quality of life and the potential role of totally implantable cochlear implants
Source: Health Expect. 2024 Apr 29;27(3):e14060. doi: 10.1111/hex.14060 (PMC11058281; doi:10.1111/hex.14060)
Supplement: Supplementary file 1 — Supporting information. [file HEX-27-e14060-s001.pdf]

Semi-structured Interview Schedule

[Introduction] We are gathering insights from cochlear implants (CI) recipients to explore the impact that having an implant has on their lives. I am a researcher at the Australian Institute of Health Innovation, and welcome your views, feelings, and experiences. We are very interested in your honest opinions, and there are no right or wrong responses. This interview should take around 1-hour but as there are a range of topics that I would like to cover, I may need to move the interview along at different points along the way. Your comfort is of utmost importance. As a reminder, you are free to pause and stop this interview at any time, without consequence. Do you have any questions so far? Can I confirm you have read the Pre-interview Information Sheet? [if not, the interviewer will go through this sheet now].

1) How has your CI made a difference to your hearing?
   (PROMPT: Examples, if required. What is the most significant difference?)

2) How does your CI impact your quality of life?
   (PROMPT: How does that compare to your quality of life before the implant?)

3) What do you think are the greatest benefits with your CI?
   (PROMPT: How do these benefits make you feel?)

4) Do you think a more discreet CI, but which works in a similar fashion to a CI, may make any of the benefits you described even more impactful?
   (PROMPT: Why/why not?)

5) What do you think are the greatest challenges or disbenefits of your CI?
   (PROMPT: How do these challenges/disbenefits make you feel? Have you ever felt stigmatised because of your CI?)

6) Do you think a more discreet CI, similar to the CI you currently use, may help overcome any of those challenges?
   (PROMPT: Why/why not?)

7) Could you imagine any downsides to a more discreet device?
   (PROMPT: Why/why not?)

8) How does your CI impact your social life, work life, and relationships?
   (PROMPT: Is the impact particularly noticeable in certain contexts? e.g., with family, friends, acquaintances, work colleagues? In groups or with individuals? In formal or informal settings? In groups or with individuals? In quiet or noisy settings? When you are nearer or further away from people? When you are listening to someone's voice or a broadcast/TV/etc.)

9) Do you think that a discreet CI device would make a difference to your social life, work life, and/or relationships?
   (PROMPT: If so, how? Why do you think that? Under what circumstances might it make the most difference?)

   9a) [If so] Would that influence your decision when choosing such a CI?

10) Before you received your CI, how did you learn about CI devices?
(PROMPT: Was it difficult to find that information? The right information? Enough information?)

11) What has your hearing health care pathway been like?
(PROMPT: Quick, fragmented, clear, supportive, shared between healthcare professionals? Was information or support provided to other family members?).
(PROMPT: How were you diagnosed with a deafness, and what was the process of implantation?)

12) What is your relationship with hearing health professionals and organisations?
(PROMPT: How did they influence you? What helped you the most, e.g., meeting facilitators, or reading testimonials of CI recipients, speaking to a supportive healthcare professional, speaking to a knowledgeable healthcare professional, being referred to the right person for your individual needs?)

13) How do you normally find or learn about new information regarding cochlear implants, implant developments, or implant updates?

14) [Wrap-up] Do you have any final thoughts or comments, particularly regarding a discreet CI device?
